# Male-specific, replicable and functional roles of genetic variants and cerebral gray matter volumes in ADHD: a gene-wide association study across *KTN1* and a region-wide functional validation across brain

**DOI:** 10.1186/s13034-022-00536-0

**Published:** 2023-01-06

**Authors:** Xingguang Luo, Xiandong Lin, Jaime S. Ide, Xinqun Luo, Yong Zhang, Jianying Xu, Leilei Wang, Yu Chen, Wenhong Cheng, Jianming Zheng, Zhiren Wang, Ting Yu, Reyisha Taximaimaiti, Xiaozhong Jing, Xiaoping Wang, Yuping Cao, Yunlong Tan, Chiang-Shan R. Li

**Affiliations:** 1 Beijing Huilongguan Hospital, Peking University Huilongguan Clinical School of Medicine, Beijing, 100096 China; 2 Department of Psychiatry, Yale University School of Medicine, New Haven, CT 06510 USA; 3 Laboratory of Radiation Oncology and Radiobiology, Fujian Medical University Cancer Hospital and Fujian Cancer Hospital, Fuzhou, 350014 China; 4 Department of Neurosurgery, The First Affiliated Hospital, Fujian Medical University, Fuzhou, 350004 Fujian China; 5 Tianjin Mental Health Center, Tianjin, 300222 China; 6 Zhuhai Center for Maternal and Child Health Care, Zhuhai, 519000 Guangdong China; 7 Shanghai Mental Health Center, Shanghai Jiao Tong University School of Medicine, Shanghai, 200030 China; 8 National Medical Center for Infectious Diseases, Huashan Hospital, Fudan University School of Medicine, Shanghai, 200030 China; 9 Department of Neurology, Shanghai First People’s Hospital, Shanghai Jiao Tong University, Shanghai, 200080 China; 10 Department of Psychiatry, Second Xiangya Hospital, Central South University; China National Clinical Research Center On Mental Disorders, China National Technology Institute On Mental Disorders, Changsha, 410011 Hunan China; 11 Department of Neuroscience, Yale University School of Medicine, New Haven, CT 06510 USA; 12 Wu Tsai Institute, Yale University, New Haven, CT 06510 USA

**Keywords:** *KTN1*, ADHD, ABCD, Cortex, Basal ganglia, Gray matter volume (GMV)

## Abstract

Attention deficit hyperactivity disorder (ADHD) is associated with reduction of cortical and subcortical gray matter volumes (GMVs). The kinectin 1 gene (*KTN1*) has recently been reported to significantly regulate GMVs and ADHD risk. In this study, we aimed to identify sex-specific, replicable risk *KTN1* alleles for ADHD and to explore their regulatory effects on mRNA expression and cortical and subcortical GMVs. We examined a total of 1020 *KTN1* SNPs in one discovery sample (ABCD cohort: 5573 males and 5082 females) and three independent replication European samples (Samples #1 and #2 each with 802/122 and 472/141 male/female offspring with ADHD; and Sample #3 with 14,154/4945 ADHD and 17,948/16,246 healthy males/females) to identify replicable associations within each sex. We examined the regulatory effects of ADHD-risk alleles on the *KTN1* mRNA expression in two European brain cohorts (n = 348), total intracranial volume (TIV) in 46 European cohorts (n = 18,713) and the ABCD cohort, as well as the GMVs of seven subcortical structures in 50 European cohorts (n = 38,258) and of 118 cortical and subcortical regions in the ABCD cohort. We found that four *KTN1* variants significantly regulated the risk of ADHD with the same direction of effect in males across discovery and replication samples (0.003 ≤ p ≤ 0.041), but none in females. All four ADHD-risk alleles significantly decreased *KTN1* mRNA expression in all brain regions examined (1.2 × 10^–5^ ≤ p ≤ 0.039). The ADHD-risk alleles significantly increased basal ganglia (2.8 × 10^–22^ ≤ p ≤ 0.040) and hippocampus (p = 0.010) GMVs but reduced amygdala GMV (p = 0.030) and TIV (0.010 < p ≤ 0.013). The ADHD-risk alleles also significantly reduced some cortical (right superior temporal pole, right rectus) and cerebellar but increased other cortical (0.007 ≤ p ≤ 0.050) GMVs. To conclude, we identified a set of replicable and functional risk *KTN1* alleles for ADHD, specifically in males. *KTN1* may play a critical role in the pathogenesis of ADHD, and the reduction of specific cortical and subcortical, including amygdalar but not basal ganglia or hippocampal, GMVs may serve as a neural marker of the genetic effects.

## Introduction

Attention Deficit Hyperactivity Disorder (ADHD) is characterized as a behavioral syndrome with core symptoms of inattention and/or hyperactivity and impulsivity. Numerous imaging studies have focused on identifying structural brain markers of ADHD. Overall, the total intracranial volume (TIV) [1–6], cortical and subcortical volumes are significantly reduced in ADHD. These regions include the frontal [prefrontal [5, 7, 8], dorsolateral prefrontal [9–12], anterior frontal [13], bilateral frontal [14], superior frontal [12, 15–17], middle frontal [12, 17, 18], medial frontal [6], inferior frontal (inferior frontal [19], orbito-frontal [12, 20], and pars triangularis [21]), and precentral [16, 22, 23]], temporal (inferior temporal [24], medial temporal [25], middle temporal [12, 26], superior temporal sulcus [18] and superior temporal [18]), parietal (postcentral [23, 27], precuneus [28], inferior parietal [4], and somato-sensory [8]), occipital (occipital [18], cuneus [17], fusiform [26], and right middle occipital [26]), limbic system (anterior cingulate [12, 29–32], left middle cingulum [17], posterior cingulate [30], amygdala [18, 29, 33], hippocampus [29, 33, 34] and thalamus [34], insula [20, 35]) and cerebellar (hemisphere [13, 36, 37] and vermis [2, 38, 39]) cortex, as well as the basal ganglia (BG) (caudate [2, 34, 40], putamen [41, 42], and pallidum [10, 41, 42]), with only a very small number of studies reporting opposite findings [25, 31, 43–45]. In particular, a number of meta-analyses showed consistent volumetric reduction in the BG [41, 42, 46, 47]. The potential biological functions of these brain regions supported that the reduction of cortical and subcortical GMVs may be implicated in the pathogenesis of ADHD.

The BG-cortical and -subcortical circuits play an important role in motivation, emotion, motor and cognitive processing [48, 49]. For instance, the cognitive/associative cortico-striatal loops involve projections from the medial and lateral prefrontal cortex, orbitofrontal cortex, anterior cingulate cortex (ACC), and temporal cortex to the BG, which via the thalamus project back to the cortex. Dysfunction of this circuit may result in executive control and other cognitive deficits, similar to those that result from damage to the prefrontal cortex [50], in ADHD. The limbic loops involve projections to the limbic subregions of the BG (nucleus accumbens and ventral pallidum) from the ventral ACC, hippocampus, and insula [51], and play a central role in reward learning, emotion, attention and saliency processing. The motor loops, involving the projections from the supplementary motor area, arcuate premotor area, primary motor cortex and somatosensory cortex to the putamen, are primarily engaged in the control of voluntary movements and motor learning. The BG circuits are heavily innervated by the dopaminergic midbrain. The medications used in the treatment of ADHD, including methylphenidate (Ritalin) and amphetamine (Adderal), are thought to block the re-uptake of dopamine to enhance dopaminergic signaling in the BG circuits [52].

Genetics contribute significantly to cerebral GMVs [53–59]. The GMVs of seven subcortical structures, including the BG (nucleus accumbens, caudate, putamen, and pallidum) and limbic system (amygdala, hippocampus and thalamus) were genome-wide studied recently, and five genetic variants that significantly influenced the GMVs of the putamen (*KTN1*, *DCC*, *BCL2L1* and *DLG2*) and caudate nucleus (*FAT3*) [54] were identified. The strongest effects were found between the putamen and rs945270 at 3’ region flanking *KTN1*. *KTN1* encodes kinectin 1 receptor, which regulates neuronal cell shape and volume [54, 60–62], and thus kinectin 1 expression may regulate brain volumes. Considering the reduction of BG GMV in ADHD, a few studies have investigated the relationship between the *KTN1* variants and ADHD. A genetic marker rs945270 in the 3′ region flanking *KTN1* showed a significant effect on the severity of hyperactivity symptoms of ADHD patients (n = 1834) and reward-related activities of the putamen in girls with ADHD [53]. We previously reported 27 *KTN1* SNPs located in four variant blocks in 5′ and 3′ flanking regions in association with ADHD across two independent family-based samples (n = 924 and 613, respectively) [63]. However, these findings were limited in that the associations among *KTN1* SNPs, *KTN1* mRNA expression in the brain, cortical and subcortical GMVs, and ADHD were not examined together in the same samples. For instance, the SNP-mRNA and SNP-GMV associations were analyzed only in healthy populations [54]; the SNP-ADHD association was analyzed only in the samples without GMV data [53]; and the GMV-ADHD association was analyzed only in the samples without any *KTN1* SNP data [64]. Thus, the interrelationships of SNPs, mRNA expression, GMVs and ADHD have remained unclear.

In the present study, we aimed to address this issue by employing the Adolescent Brain Cognition Development (ABCD) cohort as the discovery sample to examine the SNP-ADHD association. The ABCD Study is one of the largest longitudinal studies of brain development and child health in this country (n ~ 12,000), with quantitative symptom ADHD scores to serve as the phenotype. The ABCD cohort also contains data on the GMVs, providing an opportunity to investigate the interrelationships among *KTN1* SNPs, cortical and subcortical GMVs and ADHD symptom scores in the same sample. We thus aimed to identify replicable SNP-ADHD associations and explore the regulatory effects of the ADHD-risk alleles on mRNA expression and cortical and subcortical GMVs.

## Materials and methods

### Subjects

#### Discovery sample

The ABCD cohort comprised nearly 12,000 children (9–11 years old), including twin, triplet, sibling and unrelated subjects, enrolled from 21 sites across the country. The participants included children of diverse races and ethnicities, cultures, as well as socioeconomic status. The ABCD data comprised interviews, questionnaires, cognitive assessments, physical and mental health, social, emotional, environmental, behavioral and academic functions, and structural and functional brain magnetic resonance imaging (MRI) scans. All participants provided biospecimens (such as saliva) for genetic testing. The present study included only unrelated subjects (5573 boys and 5082 girls) whose relationships were confirmed both by self-report and genetic inference.

All participants were assessed with the Child Behavior Checklist for Ages 6–18 (CBCL/6–18) [65] and the T-scores of ADHD ranged from 50 to 80 and reflected the severity of ADHD symptoms. The ADHD T-scores served as the phenotype in the following SNP-ADHD association analysis. Because of the widely demonstrated sex differences in the prevalence, symptom severity, and potentially pathophysiology of ADHD, boys and girls were examined separately.

#### Replication samples

We examined three Caucasian samples for replication (Table 1). Sample #1 included 922 parent–child trios [2,757 subjects with 924 ADHD children (6–17 years old; mean 10.9 ± 2.9 years; 802 males and 122 females)] from the International Multisite ADHD Genetics (“IMAGE”) project [66]. Sample #2 included 735 parent–child trios [1,383 subjects with 613 ADHD children (6–17 years old; mean 12.3 ± 4.0 years; 472 males and 141 females)] from the “PUWMa” [Pfizer-funded University of California Los Angeles (UCLA), Washington University (WASH-U), and Massachusetts General Hospital (MGH)] genome-wide association study (GWAS) of ADHD project [67]. One or more sibling(s) in the same age range was included. Both or one parent plus two or more siblings were available to provide DNA samples. Sample #3 included 19,099 cases with ADHD (3–19 years old; 14,154 males and 4945 females) and 34,194 healthy controls (3–19 years old; 17,948 males and 16,246 females) from the sex-specific meta-analyses of ADHD GWAS by the Psychiatric Genomics Consortium (PGC) and the Lundbeck Foundation Initiative for Integrative Psychiatric Research (iPSYCH) project [68]. This “META_PGC_iPSYCH” project performed meta-analyses on one iPSYCH and 11 PGC cohorts that included “IMAGE” and “PUWMa” samples. The male and female participants of these samples were all analyzed separately.

**Table 1:** Demographic data for the discovery and replication samples

Variables	Males	Females
*Discovery sample: unrelated ABCD subjects*		
Sample size	5573	5082
Age (years)	9–11 (9.9 ± 0.6)	
Race (White/Black/Hispanic/Asian/Other)	3021/733/1118/114/587	2653/751/1021/120/537
*Replication sample #1: family-based “IMAGE” subjects*		
Sample size (922 parent–child trios)	802 offsprings	122 offsprings
Age (years)	6–17 (10.9 ± 2.9)	
Race	Europeans	Europeans
*Replication sample #2: family-based “PUWMa” subjects*		
Sample size (735 parent–child trios)	472 offsprings	141 offsprings
Age (years)	6–17 (12.3 ± 4.0)	
Race	Europeans	Europeans
*Replication sample #3: population-based “META_PGC_iPSYCH” subjects*		
Sample size (cases/controls)	14,154/17,948	4945/16,246
Age (years)	3–19	
Race	Europeans	Europeans

Children met diagnosis of ADHD on the basis of DSM-IV or ICD-10 criteria. The children in Sample #1 were free of single-gene disorders known to be associated with ADHD (e.g. fragile-X, phenylketonuria, hypercalcaemia, and thyroid hormone resistance), neurological conditions (e.g., hemiplegia, cerebral palsies, epilepsy, hydrocephalus, post-encephalitic syndromes, and sensorimotor handicaps), and psychosis. None met the criteria for autism or Asperger's syndrome. The children in Sample #2 were excluded if they were positive for any of the following: neurological disorder, concussion or other head injuries, lifetime diagnoses of schizophrenia, autism, or mental retardation. Diagnostic protocols in Sample #3 were similar to Samples #1 and 2. All children’s IQ scores were above 70. The demographic data of the three replication samples have been described in detail earlier [66–70].

### Genetic measurement

#### SNP genotyping, imputation and data cleaning

All ABCD subjects were genotyped using Affymetrix NIDA SmokeScreen Array (517,724 SNPs). Replication Sample #1 was genotyped on PERLEGEN Human600k microarray platform; Sample #2 on Illumina Human1M microarray platform, and Sample #3 on Illumina PsychChip (8,047,421 SNPs). To render the genetic marker sets consistent across discovery and replication samples, we imputed the untyped SNPs across the *KTN1* region based on the same reference panels of 1000 Genome Project and HapMap3 Project data using the program IMPUTE2 [71]. This *KTN1* region started from Chr14:54,995,382 (5’) to Chr14:55,550,419 (3’) (Genome Build 36), covering the entire open reading frame (ORF) of *KTN1*, 120 kb 5’ regulatory region flanking *KTN1* and 329 kb 3’ regulatory region flanking *KTN1*. Prior to data analysis, we applied stringent criteria to “clean up” the phenotype and genotype data, as described in detail previously [63, 72].

#### Zygosity inference

The ABCD subjects included unrelated subjects, siblings, and dizygotic and monozygotic twins or triplets. We inferred the genetic relationship (zygosity) between any pair of two subjects using the whole genome data. To determine the zygosity between subjects, we calculated the probability of identity-by-descent [P(IBD)] using the program PLINK [73]. First, we selected 3,317 independent (r^2^ = 0) SNPs from the whole genome (517,724 SNPs) based on (a) allele frequency > 0.05, (b) missing rate < 0.1, (c) family Mendel error rate < 0.05, (d) SNP Mendel error rate < 0.1, (e) Hardy–Weinberg equilibrium with p > 10^–5^, and (f) pruning to ensure independency (r^2^ < 0.2). On the basis of these selected SNPs, we calculated the [P(IBD)] to determine the zygosity. A pair of subjects with P(IBD) equal to 1.0 (0.95–1.0) were identified as monozygotic twins, P(IBD) = 0.5 (0.4–0.65) as dizygotic twins or siblings, and P(IBD) < 0.4 as unrelated pairs. In self-reported data, a pair of subjects with the same birthday (± 1 day), sex and race in the same family were reported as monozygotic twins (with possible bias); a pair with the same birthday (± 1 day) but different sex or race in the same family was reported as dizygotic twins; a pair within the same family but with different birthday was reported as siblings; and others were reported as unrelated. Self-reports may not be accurate (e.g., adoptions not identified). Thus, only those unrelated subjects confirmed both by genetic inference and self-report were included in this study.

#### Estimation of admixture degree

To quantify the degree of admixture in these subjects, we estimated the ancestry proportions for each individual with the program STRUCTURE [74]. We examined the proportions by utilizing the ancestry information content of a set of 3,330 ancestry-informative markers (AIMs). The AIMs (a) were selected from the whole-genome data by LD pruning [73] (see details in [72]); (b) differed in allele frequencies between Europeans and Africans at a genome-wide significance level (p < 10^–8^); (c) were not associated with any known mental disorder; (d) were in Hardy–Weinberg Equilibrium (p > 0.05), and (e) were completely independent (r^2^ = 0) of each other.

### Cortical and subcortical GMVs in ABCD cohort

We implemented voxel-based morphometry (VBM) to quantify the gray matter volumes (GMVs) of 52 cortical regions and six subcortical structures identified from high resolution T1-weighted images with the CAT12 toolbox (http://dbm.neuro.uni-jena.de/vbm/), following published routines [75]. VBM analysis identified differences in the local composition of brain tissue, accounting for large-scale variation in gross anatomy and location. The analysis included spatially normalizing individuals’ structural images to the same stereotactic space, segmenting the normalized images into distinct brain tissues, and smoothing the gray matter (GM) images. We used the raw images to avoid potential interference with the CAT12 preprocessing pipeline. T1-images were first co-registered to the MNI template space using a multiple-stage affine transformation during which the 12 parameters were estimated. Co-registration was performed with a coarse affine registration using mean square differences, followed by a fine affine registration using mutual information. Coefficients of the basic functions that minimized the residual squared difference (between individual image and the template) were estimated. Tissue probability maps constructed from healthy subjects were used in affine transformation, and affine regularization was performed with the International Consortium for Brain Mapping (ICBM) template space. T1 images were then corrected for intensity bias field and a local means denoising filter and segmented into cerebrospinal fluid, gray, and white matter. Segmented and the initially registered tissue class maps were normalized using DARTEL [76], a fast diffeomorphic image registration algorithm of SPM. As a high-dimensional non-linear spatial normalization method, DARTEL generated mathematically consistent inverse spatial transformations. We used the standard DARTEL template in MNI space, constructed from healthy subjects of the IXI-database (http://www.brain-development.org/), to drive the DARTEL normalization. Skull-stripping and final clean up (to remove remaining meninges and correct for volume effects in some regions) was performed with default parameters. Normalized GM maps were modulated to obtain the absolute volume of GM tissue corrected for individual brain sizes. Finally, the GM maps were smoothed by convolving with an isotropic Gaussian kernel (FWHM = 8 mm). We evaluated the GMVs separately for the right and left hemispheric brain regions.

### SNP-ADHD association analysis

We examined the associations between ADHD and a total of 1,020 imputed SNPs. A linear regression analysis was conducted in the discovery sample, in which the T-scores of ADHD served as the dependent variable, SNPs served as the independent variable, and the covariates included four dimensions of ancestry proportions (admixture degree) that corresponded to the race of White, Black, Hispanic and Asian. A family-based association test was conducted in the replication Samples #1 and #2 using the “—dfam” as implemented in PLINK; and the allele frequencies were compared between cases and controls in the replication Sample #3 using the Fisher’s Exact Test as implemented in PLINK. A SNP-ADHD association within the same sex with p < 0.05 across the discovery sample and at least one replication sample with the same association direction was taken as a replicable association. We applied a replication design, instead of the overly-conservative Bonferroni correction for multiple testing, to reduce false positives.

### *cis*-acting expression quantitative trait locus (*cis-*eQTL) analysis

We examined the potential regulatory effects of ADHD-risk variants on the *KTN1* mRNA expression in human postmortem brains in a UK European cohort (n = 138; BRAINEAC dataset) [77] and a European-American cohort (n = 210; GTEx dataset) [78] using *cis-*eQTL analysis. These subjects were free of neurodegenerative and neuropsychiatric disorders. In the UK European cohort, a total of eight cortical and subcortical brain regions were analyzed, including the prefrontal cortex, occipital cortex, temporal cortex, limbic system (hippocampus and thalamus), BG (putamen and substantia nigra) and cerebellar cortex. In the European-American cohort, a total of 11 brain regions were analyzed, including the prefrontal cortex (Brodmann Area or BA9), anterior cingulate cortex (BA24), amygdala, hippocampus, hypothalamus, BG (putamen, caudate, nucleus accumbens, and substantia nigra), and cerebellum. Normalized mRNA expression levels were compared between different alleles of each variant using t-test.

### Regulatory effect of risk variants on the TIV and subcortical GMVs

#### ABCD cohort

The total intracranial volume (TIV) and the GMVs of BG (caudate, putamen and pallidum) and limbic system (amygdala, hippocampus, and thalamus) of ABCD subjects were analyzed in relation to ADHD-risk SNPs with multiple linear regression. The covariates included four dimensions of ancestry proportions and ADHD T-scores, as well as the TIV (in the analyses of regional GMVs). We performed the analyses for left and right hemispheric areas and for males and females separately.

#### ENIGMA2 cohort (GWASMA of subcortical structures)

The TIV of 18,713 European subjects (17 CHAGE + 29 ENIGMA2 cohorts) [79] and the GMVs of BG (caudate, putamen, pallidum, and accumbens) and limbic system (amygdala, hippocampus, and thalamus) of 38,258 European subjects (14 CHAGE + 35 ENIGMA2 + 1 UKBB cohorts) [54, 80] were quantified following a standardized protocol using the brain segmentation software packages: FIRST [81] or FreeSurfer [82]. Left- and right- hemispheric GMVs were averaged in these data. All subjects were genotyped using microarray and imputed to the 1000 Genome Project genotype panels. The genetic homogeneity was assessed in each subject using multi-dimensional scaling (MDS).

We examined the potential regulatory effects of ADHD-risk variants on the TIV and regional GMVs using multiple linear regression, controlling for age, sex, 4 MDS components, TIV (for non-TIV phenotypes) and diagnosis (when applicable; most subjects were free of neurodegenerative and neuropsychiatric disorders). Males and females were not analyzed separately in this existing cohort, but sex was controlled for as a covariate.

### Regulatory effects of risk variants on cortical GMVs of ABCD subjects

The potential regulatory effects of ADHD-risk variants on a total of 104 GMVs of 52 cortical regions (right and left hemispheres separately) were analyzed using a multiple linear regression, in which the GMV of each region served as dependent variable, the SNPs served as independent variables, and the covariates included TIV, four dimensions of ancestry proportions, and ADHD T-scores. Males and females were analyzed separately.

## Results

### Replicable associations between risk variants and ADHD in males

In the discovery sample, among the 1020 cleaned, imputed SNPs, 137 and 40 SNPs were nominally (p < 0.05) associated with ADHD T-scores in males and females, respectively.

In males, 21 of these 137 SNPs were also associated with ADHD in at least one replication sample; however, only four tagSNPs were associated with ADHD in the same effect direction, reflecting true replication. These four tagSNPs were independent of each other (r^2^ < 0.05), including rs10132888 (50 kb flanking 5’ of *KTN1*) and rs10150277 (36 kb flanking 3’ of *KTN1*), rs8004764 (58 kb flanking 3’ of *KTN1*) and rs17090738 (245 kb flanking 3’ of *KTN1*). The former three were common variants with minor allele frequencies (MAF) > 0.05 and the latter one was a rare variant with MAF < 0.05. The major allele A (*f* > 0.5) of rs10132888 increased both the ADHD T-score in the discovery sample (β > 0; 9 = 0.041) and risk for ADHD in the replication sample “PUWMa” (OR > 1; p = 0.007) (Table 2). The major allele C (*f* > 0.5) of rs10150277 and the major allele T (*f* > 0.5) of rs8004764 increased both ADHD T-score in the discovery sample (β > 0; p = 0.028 and 0.020, respectively) and risk for ADHD in the replication sample “IMAGE” (OR > 1; p = 0.010 and 0.030, respectively) (Tables 5, 6, 7 and 8). The minor allele G (*f* < 0.5) of rs17090738 increased the ADHD T-score in the discovery sample (β > 0; p = 0.024) and risk for ADHD in the replication samples, including “PUWMa” (OR > 1; p = 0.015) and “META_PGC_DBS_males” (Z > 0; p = 0.003) (Table 9).

**Table 2:** p values for rs10132888-ADHD (in males) and -mRNA associations

"SNP-ADHD" association			"SNP-mRNA" association (Europeans)						
Risk allele	Discovery sample (ABCD)	Replication sample (PUWMa)	Effective allele	GTEx	BRAINEAC	GTEx	GTEx	GTEx	GTEx
				Cortex	Limbic system		BG	Cerebellum	
				Frontal	Thalamus	Hippocampus	NAc	CRBL	CRBH
A	0.041	0.007	G	0.003	4.8 × 10^–4^	0.007	1.2 × 10^–5^	0.003	0.032

Risk allele is a major allele (f > 0.5)

BG, Basal ganglia; NAc, Nucleus accumbens; CRBL, Cerebellar cortex; CRBH, Cerebellar hemisphere

**Table 3:** p values for rs10132888-TIV and -subcortical GMV associations

“SNP-TIV”		“SNP-subcortical GMV” associations (Europeans)								
Effective allele	M + F	Effective allele	M + F	M + F	M + F	M + F	M + F	M + F	M + F	M + F
			Basal ganglia							
	TIV ENIGMA2		Caudate "unrestricted"	Caudate ENIGMA2	Putamen "unrestricted"	Putamen ENIGMA2	NAc "unrestricted"	NAc ENIGMA2	Pallidum "unrestricted"	Pallidum ENIGMA2
G	0.013	A	0.004	0.016	4.3 × 10^–8^	1.2 × 10^–5^	8.9 × 10^–4^	0.033	0.005	0.014

M, male; F, female; TIV, total intracranial volume; GMV, gray matter volume; NAc, nucleus accumbens

**Table 4:** p values for rs10132888-cortical GMV associations in ABCD

Effective allele	Right posterior cingulum	Left lingual	Effective allele	Right cerebellum	Vermis
A	0.040	0.045	G	0.041	0.011

In females, none of the 40 nominal risk SNPs was associated with ADHD in the same effect direction across discovery and replication samples.

### The ADHD-risk alleles significantly decreased the *KTN1* mRNA expression in brains

All ADHD-risk alleles of the four risk variants identified in males significantly decreased (t < 0) the *KTN1* mRNA expression consistently across the frontal and occipital cortices, limbic system (hippocampus and thalamus), BG (nucleus accumbens and substantia nigra) and cerebellum (1.2 × 10^–5^ ≤ p ≤ 0.039; Tables 2, 3, 4, 5, 6, 7, 8, 9, 10), with rs10132888 demonstrating the most significant regulatory effect on mRNA expression in the nucleus accumbens (p = 1.2 × 10^–5^; Table 2). Further, the regulatory effect in cerebellum was replicable across two independent samples (BRAINEAC and GTEx), and rs10132888, rs10150277 and rs17090738 all showed significant regulatory effects in the frontal cortex.

**Table 5:** p values for rs10150277-ADHD (in males) and -mRNA associations

“SNP-ADHD” association				“SNP-mRNA” association							
Risk allele	Discovery sample (ABCD)	Replication sample (IMAGE)		Effective allele	GTEx	GTEx	BRAINEAC	GTEx	GTEx	GTEx	GTEx
					Cortex			Limbic system	Basal ganglia		
					Frontal	Frontal	Occipital	Hippocampus	NAc	SNc	Cerebellum
C	0.028	0.010		T	0.014	0.020	2.2 × 10^–4^	0.010	0.026	0.001	0.001

Risk allele is a major allele (f > 0.5). NAc, nucleus accumbens

**Table 6:** p values for rs10150277—subcortical GMV associations

Effective allele	M + F "UR"	M ABCD	M ABCD	M + F "UR"	M + F ENIGMA2	M ABCD	M ABCD	M + F "UR"	M + F ENIGMA2	M + F "UR"	M + F ENIGMA2	M ABCD	M ABCD	M + F ENIGMA2
	Basal ganglia												Limbic system	
	Caudate	Left caudate	Right caudate	Putamen	Putamen	Left putamen	Right putamen	NAc	NAc	Pallidum	Pallidum	Left pallidum	Right pallidum	Hippocampus
C	1.3 × 10^–4^	0.005	0.019	2.8 × 10^–22^	5.3 × 10^–11^	2.9 × 10^–4^	8.7 × 10^–4^	2.3 × 10^–4^	0.013	2.7 × 10^–6^	2.0 × 10^–5^	6.5 × 10^–4^	7.0 × 10^–4^	0.010

M, male; F, female; “UR”, “unrestricted” cohort; NAc, nucleus accumbens; GMV, gray matter volume

**Table 7:** p values for rs10150277—cortical GMV associations in ABCD

Effective allele	Right pars trianglaris	Left inferior temporal	Left fusiform	Right cerebellum-4/5	Left cerebelum	Right cerebellum-6	Effective allele	Right temporal pole
C	0.025	0.047	0.050	0.032	0.007	0.019	T	0.024

**Table 8:** p values for rs8004764-ADHD (in males), -mRNA, and -GMV associations

“SNP-ADHD” association			“SNP-mRNA” association		“SNP-TIV” association		“SNP-subcortical GMV” association			
Risk allele	Discovery sample (ABCD)	Replication sample (IMAGE)	Effective allele	BRAINEAC Limbic system Hippocampus	Effective allele	ENIGMA2 TIV	Effective allele	ENIGMA2		
								Basal ganglia		
								Putamen	NAc	Pallidum
T	0.020	0.030	C	0.001	C	0.010	T	0.040	0.023	0.002

Risk allele is a major allele (f > 0.5). TIV, total intracranial volume; NAc, nucleus accumbens

**Table 9:** p values for rs17090738-ADHD (in males), SNP-mRNA and SNP-subcortical GMV associations

“SNP-ADHD” association				“SNP-mRNA” association					“SNP-subcortical GMV” association				
Risk allele	Discovery sample (ABCD)	Replication sample (PUWMa)	Replication sample (META_PGC_iPSYCH)	Effective allele	GTEx	BRAINEAC	GTEx	BRAINEAC	Effective allele	M + F "UR"	M + F "UR"	Effective allele	M ABCD
					Cortex		Basal	Limbic		Basal ganglia			Limbic system
					Frontal	Occipital	NAc	THAL		NAc	Pallidum		Left amygdala
G	0.024	0.015	0.003	A	0.011	0.003	-	0.039	G	0.022	0.032	A	0.030

Risk allele is a minor allele (f < 0.5)

M, male; F, female; “UR”, “unrestricted” cohort; NAc, nucleus accumbens; GMV, gray matter volume

**Table 10:** p values for rs17090738—cortical GMV associations in ABCD

Effective allele	Left pars triangularis	Left postcentral	Left inferior parietal	Right inferior parietal	Left angular	Effective allele	Right rectus
G	0.024	0.028	0.013	0.009	0.033	A	0.010

### ADHD-risk variants significantly regulated TIV and subcortical GMVs

The ADHD-risk alleles of rs10132888 (p = 0.013; Table 3) and rs8004764 (p = 0.010; Table 8) significantly decreased the TIV.

The ADHD-risk alleles of all four variants significantly increased the GMVs of the BG (caudate: 1.3 × 10^–4^ ≤ p ≤ 0.019; putamen: 2.8 × 10^–22^ ≤ p ≤ 0.040; nucleus accumbens: 2.3 × 10^–4^ ≤ p ≤ 0.033; and pallidum: 2.7 × 10^–6^ ≤ p ≤ 0.032) consistently across multiple independent samples (Tables 3, 6, 8 and 9). Putamen GMV was most significantly regulated by the *KTN1* ADHD-risk alleles. The ADHD-risk alleles of rs10132888 and rs10150277 most significantly increased the putamen GMVs across two (4.3 × 10^–8^ ≤ p ≤ 1.2 × 10^–5^; Table 3) and three (2.8 × 10^–22^ ≤ p ≤ 3.0 × 10^–4^; Table 6) independent samples, respectively. The ADHD-risk allele of rs10150277 also significantly increased the hippocampus GMV (p = 0.010; Table 6). In contrast, the ADHD-risk allele of rs17090738 significantly reduced the amygdala GMVs (p = 0.030; Table 9). None of the other regulatory effects on subcortical GMVs were significant.

### ADHD-risk variants significantly regulated the cortical GMVs

The ADHD-risk variants significantly regulated the cortical GMVs in the frontal (gyrus rectus and inferior frontal gyrus, pars triangularis), temporal (superior temporal pole), parietal (postcentral, inferior parietal and angular gyri), and occipital lobes (fusiform and left lingual gyri), as well as the limbic system (posterior cingulate cortex), and cerebellum (hemisphere and vermis). Specifically, the ADHD-risk allele of rs10132888 increased (β > 0) the GMVs of the right posterior cingulate cortex (p = 0.040) and left lingual gyrus (p = 0.045), but decreased (β < 0) the GMVs of the right hemisphere (p = 0.041) and vermis (p = 0.011) of the cerebellum (Table 4). The ADHD-risk allele of rs10150277 increased the GMVs of the right inferior frontal cortex, pars triangularis (p = 0.025), left inferior temporal cortex (p = 0.047), left fusiform gyrus (p = 0.050) and cerebellum (0.007 ≤ p ≤ 0.032), but decreased the GMV of the right superior temporal pole (p = 0.024) (Table 7). The ADHD-risk allele of rs17090738 increased the GMVs of the left inferior frontal cortex, pars triangularis (p = 0.024), left postcentral cortex (p = 0.028), left inferior parietal cortex (p = 0.013) including the angular gyrus (p = 0.033), and right inferior parietal (p = 0.009) cortex, but decreased (β < 0) the GMV of the right rectus gyrus (p = 0.010) (Table 10).

## Summary of the results (Table 11)

**Table 11:** Summary of directions of associations with ADHD-risk alleles

Phenotype	ADHD-risk	mRNA		TIV	Subcortical GMV				Cortical GMV	
Class					Basal ganglia		Limbic system		Cortex	Limbic system
Regions							HIPP	AMYL		
Cohorts		BRAINEAC	GTEx	ENIGMA2	ENIGMA2	ABCD	ENIGMA2	ABCD	ABCD	ABCD
↑: increase	X				X	X	X		F,P,T,O,C	PCC
↓: decrease		X	X	X				X	F,T,C	

TIV, total intracranial volume; GMV, gray matter volume; HIPP, hippocampus; AMYL, amygdala

F, frontal; P, parietal; T, temporal; O, occipital; C, cerebellar; PCC, posterior cingulate cortex

Four *KTN1* variants significantly regulated risk for ADHD in males, consistently across discovery and replication samples. All of the ADHD-risk alleles significantly decreased the *KTN1* mRNA expression in all brain regions examined (in both BRAINEAC and GTEx), most prominently in the BG. The ADHD-risk alleles significantly increased the GMVs (in both ENIGMA2 and ABCD) of the BG and hippocampus (in ENIGMA2), but reduced the GMV of the amygdala (in ABCD) and the TIV (in ENIGMA2), and increased or decreased the cortical GMVs (in ABCD) as well.

## Discussion

We identified four replicable, independent risk variants for ADHD specifically in males, each located at the 5′ (rs10132888) or 3′ (rs10150277, rs8004764 and rs17090738) flanking region. Variants rs10132888 and rs10150277 have also been reported earlier in association with ADHD risk [63]. All four risk alleles had significant biological functions, including regulation of *KTN1* mRNA expression, TIV, and subcortical and cortical GMVs, supporting the roles of the *KTN1* variants in the development of ADHD in males. These findings also suggested potential sex difference in the genetic bases of ADHD.

Globally, the ADHD-risk alleles decreased the TIV, consistent with numerous previous findings [1–6]. Regionally, this set of ADHD-risk *KTN1* alleles regulated the mRNA expression, and/or GMVs widely across brain regions, including both cortical and subcortical structures and the cerebellum. The affected cortical structures included those in the frontal, temporal, parietal and occipital lobes as well as the limbic system, including the posterior cingulate cortex. The affected subcortical structures included primarily the BG—caudate, putamen, nucleus accumbens and pallidum. In the BG, the GMV of the putamen was most significantly and reliably affected by this set of *KTN1* ADHD-risk alleles, consistent with the report that *KTN1* was the most significant gene regulating the GMV of putamen [54], and the putamen GMV was the most significant regulator among all brain regions mediating the association between *KTN1* and ADHD [83].

A number of meta-analyses have demonstrated volumetric reduction of the BG and the putamen, in particular, as a structural marker of ADHD [41, 42, 46, 47]. Here, we demonstrated that this set of ADHD-risk alleles significantly increased BG (especially putamen), hippocampus, and some cortical GMVs. Apparently, in ADHD-risk allele carriers, the protective effects of BG and cortical GMVs were weaker than the risk effects of these alleles, so that the overall ADHD risk was still significantly higher, as observed in the present study. These findings suggest that this set of alleles and BG/cortical GMVs independently affect the ADHD risk; that is, BG/cortical GMVs did not mediate the ADHD risk effects of this set of *KTN1* alleles. However, we also demonstrated that this set of ADHD-risk alleles significantly decreased other cortical (right superior temporal pole, right gyrus rectus, cerebellar hemisphere and vermis) and subcortical (amygdalar) GMVs (Table 11). Thus, both the ADHD-risk alleles and the volumetric reduction of the latter brain regions may contribute to the ADHD risk in the same effect direction, and these regions might support the risk effects of these *KTN1* alleles.

The regional GMVs reduced by this set of risk alleles have been implicated in the development of ADHD symptoms, including inattention (cerebellar cortex) [84], altered emotional processing and impulsivity (amygdala) [85], dysfunctional social cognition (superior temporal cortex) [86], and personality (gyrus rectus) [87], potentially in support of a causal role of these ADHD-risk alleles in the development of ADHD and in consistence with the widely-reported findings about reduced regional volumes in ADHD. In particular, the cerebellar hemisphere and vermis may be implicated through the inferior fronto-striato-cerebellar circuit, central to working memory, attention, and emotional function in ADHD [84].

Notably, the most significant and consistent regulatory effects of this set of ADHD-risk alleles on regional GMVs were observed for the BG. However, as discussed above, BG GMVs did not mediate the ADHD risk effects of this set of *KTN1* alleles. Therefore, the exact mechanisms underlying the effects of these ADHD-risk alleles on BG GMVs remain to be clarified. We provided two potential hypotheses. Firstly, the cortical volume reduction, as observed here for some brain regions, may decrease the neurotransmission in the “cortico-BG-thalamo-cortical” loop. As a compensatory response to the reduction of excitatory glutamatergic cortical projections to the BG, the volume of BG may expand to maintain the neurotransmission within the loop, leading to the observed association between the ADHD-risk alleles and higher BG GMVs. The expanded BG volume did not mediate the risk effects of this set of *KTN1* alleles on ADHD, but rather reflected the consequences of the compensation response to expression of ADHD-risk alleles in the cortical structures. Broadly in support of this hypothesis are findings that boys relative to girls [88] and ADHD relative to neurotypical children [89] showed higher impulsivity and cortical striatal functional connectivity. Alternatively, other genetic or environmental factors might elevate BG GMVs. To compensate the BG volume increase, the volume-controlling alleles, such as the ADHD-risk *KTN1* alleles, might be stimulated to express phenotypes that included the BG volume reduction and ADHD symptoms. The compensated BG volume reduction caused by the ADHD-risk *KTN1* alleles usually does not completely restore the expanded BG volume and neurotransmission induced by other dominant genetic and environmental factors. This hypothesis well interpreted the associations between the ADHD-risk alleles and the expanded BG volume (Table 11). Again, the expanded BG volume did not mediate the risk effects of these *KTN1* alleles on the ADHD risk, but might an inducer of the expression of ADHD. Besides these two hypotheses, other mechanisms to interpret our findings cannot be excluded.

Finally, the ADHD-risk alleles decreased the *KTN1* mRNA expression but increased BG and some cortical GMVs, indicating that lower *KTN1* mRNA expression was not necessarily associated with lower GMVs. This may reflect incongruence between mRNA and protein expression levels in these structures. Furthermore, the ADHD-risk allele of rs8004764 decreased the GMVs of the right hemisphere and vermis of cerebellum, but the risk allele of rs10150277 increased the GMVs of the cerebellum, suggesting that cerebral GMVs represent a multi-genic phenotype.

In summary, we identified a set of significant, functional, and robust risk *KTN1* alleles for ADHD. These alleles increased the risk for ADHD, decreased the *KTN1* mRNA expression in the brain, and reduced cerebellar, some cortical, and amygdalar GMVs. Studies are warranted to further investigate the roles of these *KTN1* risk alleles in the pathogenesis of ADHD.

## Data Availability

The ABCD dataset was obtained from https://nda.nih.gov/abcd/. Other datasets used for the analyses described in this manuscript were obtained from dbGaP at http://www.ncbi.nlm.nih.gov/sites/entrez?Db=gap. dbGaP Study Accession: phs000016.v2.p2 and phs000358.v1.p1.
